# The lived experiences of HIV-positive women in rural Zimbabwe: A qualitative focus group study

**DOI:** 10.4102/safp.v66i1.5823

**Published:** 2024-03-18

**Authors:** Limkile Mpofu, Makombo Ganga-Limando

**Affiliations:** 1 Department of Health Studies, Faculty of Human Sciences, University of South Africa, Pretoria, South Africa

**Keywords:** stigma and discrimination, rural women, Interpretative Phenomenological Analysis (IPA), HIV and AIDS, rural Zimbabwe, meanings attached, lived experiences, framework

## Abstract

**Background:**

The study explored and described the meaning attached to the lived experiences of women living with human immunodeficiency virus (HIV) in the rural context of Zimbabwe. Stigma and discrimination negatively impact one’s ability to perform the expected social roles, the quality of life, and the efforts to prevent the spread of HIV and acquired immunodeficiency syndrome (AIDS) and reduce HIV-related mortality. Thus, the study aims to understand the meaning attached to the lived experiences of HIV-positive women living in rural areas or villages of Matabeleland South province in Zimbabwe.

**Methods:**

The study used a qualitative, descriptive, and exploratory design. Four focus group discussions were conducted with 24 HIV-positive rural women living in Matabeleland South province, Zimbabwe. An Interpretative Phenomenological Analysis (IPA) was adopted to explore and describe the meaning attached to the lived experiences of women living with HIV.

**Results:**

Two interconnected themes were identified in the analysis with their sub-themes. These were: (1) struggle for social belonging, with subthemes – loss of social belonging and reduced access to community-based empowerment opportunities and (2) struggle for maintaining the quality of life with subthemes – lack of need-based community healthcare and food insecurity.

**Conclusion:**

Being a woman living with HIV in rural Zimbabwe means a perpetual struggle to maintain one’s humanness and quality of life.

**Contribution:**

This study’s results will support the efforts of the Zimbabwean government to improve the quality of life of HIV-positive women living in rural areas.

## Introduction

Even after 40 years since the outbreak of the human immunodeficiency virus (HIV) pandemic, more than 40 million people have died of acquired immunodeficiency syndrome (AIDS) globally.^1^ Human immunodeficiency virus and AIDS continue to spread worldwide. Quinn reports that in 2020, 1.5 million new cases of HIV infection occurred. Such an increase in new cases of HIV infection serves to highlight that the control and prevention of HIV remain daunting challenges globally.^2^ The current state of the global HIV and/or AIDS pandemic confirms the importance of government interventions to prolong life expectancy, increase the quality of life among women already infected with the disease, and prevent new cases. However, HIV-related stigma and discrimination continue to obstruct attempts to prevent new infections and engage people in HIV treatment, care, and support programmes.^3^. In Zimbabwe, out of 1.3 million people living with HIV (PLHIV), more than half of that population are females of child-bearing age.^4^ Logie et al.^5^ posit that HIV is an epidemic fuelled by racial, gender, and class inequities. Antiretroviral treatments saw the shift of HIV to a manageable chronic condition; however, these people may still experience adverse social effects and unfair treatment.^6^ Thus, the global AIDS response success relies on identifying effective interventions to reduce stigma and discrimination.^3^ Such interventions include measures to reduce the HIV-related stigma, which other research has recognised as preventing many people from seeking appropriate treatment.

Human immunodeficiency virus-related stigma and discrimination are multidimensional and socially constructed, consequently affecting the social and living experiences of PLHIV. The social conditions for HIV-positive women living in rural Zimbabwe (e.g., a more homogeneous social environment with people sharing the same social values and beliefs and social role expectations) are different from those of their counterparts living in urban areas.^7^ These conditions can influence how these women react to stigma and discrimination and their meanings to their living experiences.^8^

Culturally, in Zimbabwe, decision-making is for men, and women must yield and submit to their decision-makers.^9^ Women’s role in society has been culturally stereotyped even though it is vital for women to make their own choices. Halli et al.^10^ studies report that stigma and discrimination towards PLHIV often occur within families by parents, siblings, relatives, or in-laws. This means that personal opinions of PLHIV’s families are a significant predictor of behaviour, even though other sources may cause subjective normative beliefs to shift.^11^ A person’s perception of the opinions of significant others concerning a particular action affects subjective norms. Subjective norms are beliefs a group will approve and support a specific behaviour.^12^ For example, in the case of rural Zimbabwean women living with HIV, subjective norms from the support group or peers, family, or society could be thoughts such as feeling suspicious of people’s intentions towards the person living with HIV.

A clearer understanding of rural women (living with HIV), their attitudes, and beliefs is needed to address underlying drivers of HIV-related stigma. Such understanding requires eliciting these women’s characteristics and behaviours, so that the roots of stigma can be understood and addressed most effectively.^10^

In this study, the researchers used social constructivism as a philosophical foundation to answer the research question. Social constructivism ascertains that reality is constructed through human interaction (ontology) and that knowledge is socially and culturally constructed (epistemology).^13^ According to Creswell,^13^ social constructivism is based on the following three main assumptions: human beings construct meanings as they engage with the world they are interpreting; human beings engage with their world and make sense of it based on their historical and social perspectives; and the basic generation of meanings is always social, arising in and out of interaction with a human community.^13^

As social constructivists, the researchers also believe that multiple realities are constructed through individuals’ lived experiences and interactions with others (ontological assumption) and that reality is co-constructed between the researcher and the participants and shaped by individual experience (epistemological assumption).^14^ Cuthbertson et al.^15^ argue that social constructivism focuses on the process of describing and explaining the socially constructed world. In social constructivism, the researcher considers the interaction between individuals, groups, and societies in their social contexts through language.^16^

The choice of social constructivism was based on the understanding that women living with HIV developed new meaning about the realities of being HIV-positive and accumulated knowledge through their lived experiences and interactions with others in their social, cultural, and socio-economic context of rural areas in Zimbabwe. Consequently, the researchers can interact with them to depict and interpret these realities and their meaning. With the understanding of stigma and discrimination being socially constructed, the researchers viewed social constructivism as more appropriate to guide this study. Therefore, this study aimed to understand the meaning of being a woman living with HIV in the rural context of Zimbabwe. As the women face the challenges of living with HIV and AIDS, they may have certain perceptions of their acceptability as living with HIV in their community. Such perceptions from their lived experiences would increase the understanding of the new realities of living with HIV in the rural context of Zimbabwe.

## Research methods and design

### Study design

The researchers used the Hermeneutics Phenomenological design because phenomenological research design is based on the understanding that experience is a valid, rich, and rewarding source of knowledge.^17^ Phenomenology enables researchers to construct realities as lived and described by the participants.^18^ According to Van Manen,^19^ phenomenology allows researchers to generate data on the informants’ experiences and lived shared experiences and derive meaning from these descriptions. Hermeneutics uses lived experiences to understand better the social, cultural, political, or historical context in which those experiences occur.^18^

### Setting

The researchers conducted the study in Matabeleland South province, one of the provinces in Zimbabwe. Matabeleland South province shares borders with South Africa and four ports of entry (Beit Bridge Border Post, Plumtree, Maitengwe, and Mphoengs). The province is divided into six districts.

The relatively higher prevalence of PLHIV (21.6%) and the social and cultural homogeneity of the population informed the selection of this province.^20^ The population shares the same culture and language. The geographical situation was also crucial for this study as most males in these bordering areas migrate to South Africa for employment.

### Study population and sampling strategy

The study population included all women living with HIV in rural Matabeleland South province.

All women living with HIV in rural areas or villages of Matabeleland South province were eligible to participate in the study. In Zimbabwe, PLHIV are cared for at the community level by community health workers attached to the respective district health. Each community health worker keeps a register of the PLHIV under their care and records of the care provided.

The researchers used non-probability purposive sampling techniques to select the participants. The researchers chose participants who best articulated their experiences with the phenomenon of interest.

The researchers used the following inclusion criteria to select eligible participants:

1. be an adult woman (18 years and above) living with HIV
2. be tested HIV-positive for at least 1 year from the date of the data collection
3. be living permanently in the village for at least 1 year after being tested HIV-positive
4. be able to give a good account of herself.

The researchers excluded all women living with HIV who were on treatment for any psychiatric condition from the study.

The researchers identified eligible participants from the community health workers’ registers. The researchers sent an individual invitation to the eligible participants via the community health workers. The researchers met individually with those who responded to the invitation, where they received further information about the study. At the end of the meeting, each participant was asked if she was willing to participate in the study. Twenty-four women living with HIV were included in the sample. The researchers selected the participants from six villages (one village per district) of Matabeleland South province.

### Data collection

In this study, the researchers used focus groups to collect data. The participants signed a non-disclosure document before the focus group discussion. As a result of the topic’s sensitivity, the researchers allowed the participants to select the methods they felt more comfortable with. The researchers conducted four focus groups with six participants each.

The researchers used interview schedules to guide data collection. The schedule was divided into two sections. The first section dealt with general information. The second section contained one open grand tour question (an opening question designed to describe the phenomena in the person’s own words) to allow themes to emerge and avoid premature closure.^19,21^

For these focus groups, the discussions were guided by the following question:

Tell me about your life experiences as a woman living with HIV in this village, and What does it mean to you as a woman living with HIV in this village? (p. 2)

Probing questions focused on the meaning of the lived experiences.

The researchers obtained informed consent and ensured voluntary participation. The study participants signed written informed consent forms and non-disclosure documents before the interview commenced. After receiving the signed consent form from the participants, the researchers also asked for permission to use the digital voice recorder from the participants to record the interviews. The researchers collected data over 6 months. Focus groups were conducted in venues selected by the participants. Participants of focus groups opted for venues at the nearest clinics. The participants used these clinics to collect their antiretroviral drugs and attend support group meetings for PLHIV. The researchers secured the focus group venues with the support of the community health workers.

The researchers offered the participants the choice to express themselves in English or Ndebele (the local language spoken in the province). They all opted for English with the option to express themselves in Ndebele when needed. This was not a problem as the first author also speaks the local language as a native of the province. Focus groups lasted an average of 90 min. Focus group interviews were audiotaped. The researchers reached data saturation after three focus group study participants were interviewed. However, the researchers interviewed one more focus group to ensure no new information and to declare data saturation.

The researchers transcribed the recorded data (verbatim) 24 h after each focus group discussion. The transcribed transcripts were double-checked by a local university researcher fluent in Ndebele and English. Data redundancy was monitored, ensuring the transcripts’ accuracy and coding. The final report of the findings was submitted to the same local expert for verification and confirmation of themes. This last process equals the cross-validation of data, consistent with Interpretative Phenomenological Analysis (IPA).^19^

### Data analysis

This study used IPA to guide data management and analysis. The search for meaning is central to the IPA analysis. Such a process involves the researcher engaging in an interpretative relationship with the transcript.^19^ In this study, the researchers used the simplified version of the steps presented by Cresswell.^14^ It includes six main steps: (1) the researchers’ description of their own experience of the phenomenon; (2) the researchers develop a list of significant statements from the participants’ description; (3) grouping of the multiple reports into themes; (4) textual description of the experience and inclusion of verbatim examples; (5) structural description or the description of how the encounter happened; (6) writing of a composite report of the phenomenon incorporating the textural and structural stories.^14^

### Scientific rigour

Credibility, dependability, conformability, and transferability ensured the data quality was trustworthy.^18^

Credibility was ensured through prolonged participant interaction, member checking, data triangulation, and purposefully recruiting participants who met the study’s inclusion criteria. The researchers had extended interactions with the participants that lasted 6 months in the field to collect data. In member checking, the researchers validated participants’ responses by repeating sentences to affirm what participants were trying to communicate. Data triangulation was performed by combining data from the audiotapes and the field notes. This ensured that non-verbal cues that the audiotape could not capture were recorded in the field diary. This was done effectively through concurrent data collection and transcription.

The researchers ensured transferability by thoroughly describing the setting and methodology of the study. On the other hand, dependability was guaranteed by giving the raw data to an independent coder.

Conformability was secured by submitting the research proposal for scientific and ethical reviews to the Department of Health Studies Health Research Ethics Committee of the University of South Africa and the Zimbabwe Medical Research Council. An appropriate emotional distance between the researchers and informants was also kept to avoid influencing the findings. Most importantly, data were coded and recoded several times and compared with the themes identified by the independent coder.

### Ethical considerations

The researchers observed all the ethical principles of human objects, and the study received ethical approval from the Research and Ethics Committee of the University of South Africa (UNISA) (HSHDC/847/2018). Permission was also granted by the Medical Research Council of Zimbabwe (MRCZ) with approval reference (MRCZ/A/2398) before the researchers commenced data collection. The researchers maintained the privacy and confidentiality of the information shared by participants. All data sources were well protected. Anonymity for the participants meant that their names would neither be used nor referred to during and after data collection. The researchers only used pseudonyms (codes) and maintained participants’ privacy during data collection. The participants were recruited for voluntary participation in the study. Participants who met the inclusion criteria were identified from community health workers, as these were the point of entry. They were informed in advance that the interviews would be audio-recorded. The participants’ autonomy was respected. Before participating, the women were given information about the study in their chosen settings, and their role in participation was explained. Those who agreed to participate were given consent forms to sign to confirm their willingness to participate in the study.

## Results

Two themes and four sub-themes were derived from the meaning women living with HIV ascribed to their experiences. The themes were: (1) the struggle for social acceptance and (2) the struggle to maintain the quality of life and well-being. Table 1 summarises the two themes with the related sub-themes.

**TABLE 1:** Meaning attached to the lived experiences of women living with human immunodeficiency virus.

Themes		Sub-themes
1.	Struggle for social acceptance	1.1 Loss of social belonging 1.2 Reduced access to community-based empowerment opportunity
2.	Struggle to maintain the quality of life	2.1 A lack of need-based community health 2.2 Food insecurity

From the participants’ descriptions of the meaning attached to their experiences, *being a woman living with HIV in rural Zimbabwe means a perpetual struggle to maintain one’s humanness and quality of life.*

### Theme 1: Struggle for social acceptance

The struggle for social acceptance refers to the views of women living with HIV regarding their social status and rights as members of their communities following the disclosure of their HIV-positive status.

The given theme is derived from two sub-themes: (1) loss of social belonging and (2) reduced access to community-based empowerment opportunities. From the views expressed by women living with HIV in rural Zimbabwe, *it was deduced that being a woman living with HIV in rural Zimbabwe means a struggle for social acceptance due to the loss of social belonging and reduced access to community-based empowerment opportunities in their communities.*

#### Sub-theme 1.1: Loss of social belonging

Loss of social belonging as a sub-theme referred to the views of women living with HIV regarding social interaction with significant others and using the available social support system in their community.

Social interaction with significant others and utilising the existing social support system were viewed as an expression of the sense of belonging to the community. Participants defined themselves as social beings interacting with significant others and fully utilising the social support system when needed to express social belonging.

However, the social prejudice and discrimination perpetrated against women living with HIV by significant others excluded them from enjoying their rights of social belonging. For women living with HIV in rural Zimbabwe, their HIV-positive status made them lose their social identity as community members. These views emerged from the discussions of all four focus groups. It was best illustrated with this narrative from the first focus group:

‘We were taught to interact freely with others since our birth. This interaction is proof of our social identity as members of this community. It makes one feel that she is a community member with full rights recognised by our culture. But everything changed with our HIV-positive status. People, including your own close family members, distance themselves from you. Becomes difficult for PLHIV, specifically for us women.’ (FDG1, 45 years old, female, ECD teacher)

For women living with HIV in rural Zimbabwe, the loss of social belonging also included the exclusion from the existing social support system. They were not accepted or allowed to use the existing social support system available in the community where they lived:

‘Being HIV-positive woman means everything terrible that you can think of. Before testing positive for HIV, we enjoyed everything in this community. We were recognised and considered as full members of the family and the village. We received support from everybody in the family and community. We were invited to join all the social activities and play active roles. With our HIV-positive status, we no longer get support from our families and communities. Social support is critical in assisting you in building the confidence to conquer anything. But now … we are excluded from using all those resources.’

‘Besides, you are nothing if you don’t have a male child in this community. To our community, the land belongs to men. If you are unmarried and have no men around you, you don’t have a future in our community. Yes, my sister [*referring to the researcher*], this is what it means to be a woman living with HIV in this community.’ (FDG2, 49 years old, female, farm worker)

#### Sub-theme 1.2: Reduced access to community-based empowerment

Reduced access to community-based empowerment opportunities as a sub-theme–described the experiences of women living with HIV regarding their participation in community-based capacity-building training. These trainings were designed to empower women through skills development in various areas.

After disclosing their HIV status, women were seldom invited to attend those training:

‘You know, we used to attend many trainings organised to empower women in the community. We applied the skills learned to achieve financial independence and fulfil our basic needs. We also applied the skills learned to establish our small businesses in the communities. Oh yes, we spend the income generated from these activities on food, health, and education. With our HIV-positive status, we are no longer invited to these trainings.’ (FGD3, 39 years old, female, entrepreneur [hairdresser])

The reduced access to community-based empowerment opportunities also included support for continuing education, as transpired in the discussion from FG 4:

‘You know the role of education for women in this country. The financial gains from educating girls are huge. But, the gender gap in education is still significant. Unfortunately, once you are tested HIV-positive as a girl, no one wants to spend money on your education. It is unfortunate. For some members of our families and communities, HIV is still a death sentence.’ (FGD4, 27 years old, female, does craftswork)

### Theme 2: Struggle to maintain the quality of life

Struggle for preserving the quality of life as a theme referred to the views women living with HIV held about meeting their health and basic needs:

The above-mentioned theme is derived from two sub-themes: (1) a lack of need-based community healthcare and (2) food insecurity. From the views expressed by women living with HIV in rural Zimbabwe, *it was deduced that being a woman living with HIV in rural Zimbabwe means a struggle to maintain a quality of life due to the lack of need-based community healthcare and food insecurity in their communities.*

#### Sub-theme 2.1: Lack of need-based community healthcare

Lack of need-based community healthcare as a sub-theme refers to the views of women living with HIV regarding the availability of healthcare services to maintain their quality of life within their social context.

For women living with HIV in rural Zimbabwe, the existing healthcare services do not address their specific health needs as rural PLHIV. This situation influences how they view themselves as women living with HIV:

‘You know, we need regular health check-ups as PLHIV. The existing services are far away, but we have problems even accessing those services. We need a door-to-door medical check-up. The health workers/caregivers who go around do not provide information on the importance of living healthy with HIV. They do not know how to deal with us and reduce HIV-related stigma.’ (FDG2, 40 years old, female, local boarding school cook)

#### Sub-theme 2.2: Food insecurity

Food insecurity as a sub-theme refers to the views of women living with HIV regarding the impact of being HIV on food security within their social context.

For women living with HIV in rural Zimbabwe, HIV-positive status means food insecurity. Social discrimination has been particularly singled out as the primary reason for food insecurity:

‘We all need to have enough food in our households to maintain our quality of life. This is so important with our health status. But, the discrimination we suffer daily makes it difficult for one to continue the economic activities she was doing before being tested HIV-positive.’ (FGD3, 41 years old, female, agricultural worker)

**FIGURE 1:**
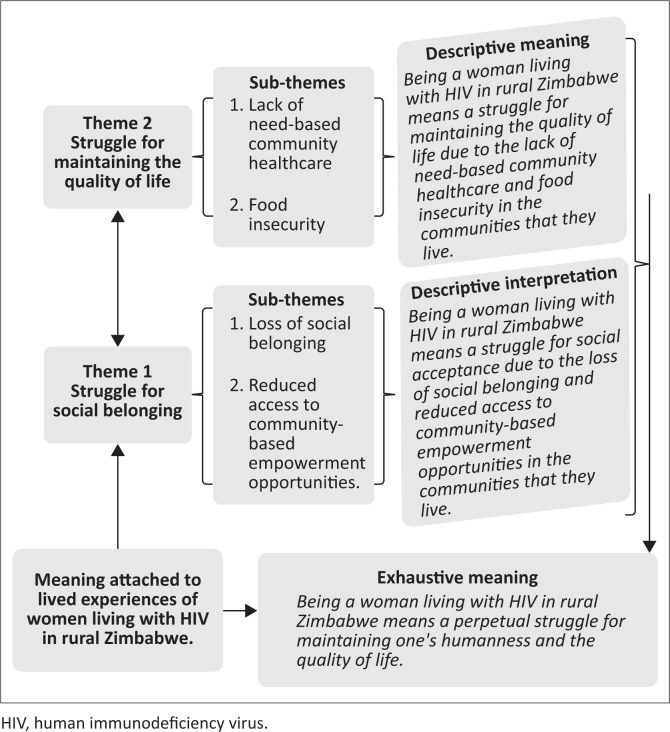
Framework of the meaning attached to the lived experiences of women living with human immunodeficiency virus in rural Zimbabwe.

The researchers used the findings to develop a framework of the meaning attached to the lived experiences of women living with HIV in rural Zimbabwe (see Figure 1).

## Discussion

The study aimed to explore the meaning attached to being a woman living with HIV in the rural context of Zimbabwe. One of the arguments of this study, as articulated in the research problem statement, was the limited understanding of how women living with HIV view their new realities of being HIV-positive and their accumulated lived experiences within the rural socio-cultural context of Zimbabwe.

The participants expressed some concern regarding the importance of land. This is because the land reforms in Zimbabwe continue to apply inheritance and land allocation rules. This widens the discrimination act against women and makes single, married, divorced, and widowed women particularly vulnerable rural women, as they lose the land upon the death of the man in whom the land is registered. In Zimbabwe, women are regarded as perpetual minors through the eyes of customary law.^22^ Women are either under their father’s authority or their husbands when they marry. Thus, marriage is highly esteemed, a norm, and a priority in life; women do all means to get attached to a man in marriage. Marriage is an expectation that society looks towards seeing from a grown-up woman.^23^ Such hope puts pressure on women who want to get married at whatever cost lest they become social outcasts. Therefore, in this study, the social prejudice and discrimination perpetrated against women living with HIV by significant others excluded them from enjoying their rights of social belonging.

Traditionally, getting married is encouraged, exposing young women to HIV and AIDS. This meant that the specific health needs of unmarried young women and adolescent girls were not met. In this study, women complain about the inadequacy of the existing healthcare services, which do not address their specific health needs as rural PLHIV. The researchers have also observed that Zimbabwe’s social environment devalues women as they cannot negotiate and decide their sexuality, making women unable to seek and enjoy good health. This is also reported by Mahamboro et al.,^24^ who posit that such discriminatory behaviour prevents women from seeking and enjoying good health as they feel their social environment is degrading and devaluing their social worth.

In this study, the women lament the decline in the traditional cultures as this was the only way they would have full rights as community members. Previous studies have also reported stigma and discrimination as negatively impacting healthcare access and health outcomes, which, according to Stangl et al.,^3^ are associated with stress, depression, and lower perceived quality of life among PLHIV. Logie et al.^25^ posit that, among HIV-positive people, those who have experienced HIV-related stigma have had higher mental and physical illness levels, hence struggling to maintain the quality of life due to discrimination.

According to a United Nations organisation for women, so deep-rooted is perpetual minors’ teaching that men have difficulty acknowledging female individuals as equals.^26^ Such teachings promote HIV-related stigma and discrimination as it reflects a struggle to maintain the quality of life due to discrimination; as seen in this research, the community perceptions that women are minors reinforce the socio-cultural attitude of the community as this attitude limits females from reaching out for safe sexual reproductive health, making them susceptible to HIV and AIDS.

Moreover, the HIV stigma and discrimination were experienced by the participants within their own families as reflected in a range of discriminatory and stigmatising attitudes and behaviours of family members. The women felt excluded from the social systems within their community, consistent with the previous study’s results reported by Halli et al. 2017.^10^ Thus, the struggle for social belonging was caused by social stigma as the women under investigation felt some loss of social belonging to their close family ties. Goodwin and colleagues also emphasise that the fear of internal stigma impacts personal and social relationships.^27^ For example, the community feared contracting HIV through physical contact or interactions, and this fear facilitated discriminatory and stigmatising attitudes and behaviours towards participants. This made the rural women living with HIV feel the loss of social belonging to their close family ties, who spread incorrect or misleading information about HIV among other family members, leading to stigma and discrimination towards these women living with HIV. This is consistent with the studies by Fauk et al.,^28^ who reported that the spread of incorrect information by significant others influenced family members, neighbours, or community members.

The focus groups here also revealed that there was reduced access to community-based empowerment opportunities, depriving the women of support for continuing education. Participants stressed the importance of social support within their community as transpired in their discussion. Besides, as mentioned above, in rural Zimbabwe, land is a source of income. Thus, land as the source of income in rural areas means that if the rural women living with HIV have no income, the HIV-related stigma and the erosion of social support will compromise the ability of rural women living with HIV and/or AIDS to secure food for their households. And this has adverse health outcomes for the rural women living with HIV. This is consistent with the studies by Verhey and colleagues, who added that stigmatisation creates a dynamic where the seropositive woman is often being blamed and punished in the form of intimate partner violence (IPV).^29^

### Limitations of the study

The data gathering probing for strategies did not include the views of other rural women living in other parts of Zimbabwe. The researchers are aware that the Ndebele’s socio-economic conditions in Matabeleland South might differ from those of rural women in Harare or any other province in Zimbabwe. For these reasons, the generalisability of the study findings is limited. These findings, therefore, need to be confirmed in different groups of rural women living with HIV in other provinces in Zimbabwe.

### Implications or recommendations

The findings of this study addressed our limited understanding of how women living with HIV view their new realities of being HIV-positive and their accumulated lived experiences within the rural socio-cultural context of Zimbabwe by providing the framework (see Figure 1). As illustrated in Figure 1, this framework included the themes, sub-themes, and the specific and descriptive meaning of the emerged experiences.

The meaning participants ascribed to being a woman living with HIV in rural Zimbabwe was closely linked to their lived experiences within the social, cultural, and socio-economic context in which they live. It means that women living with HIV in rural Zimbabwe shared the same experiences. Despite the negative lived experiences, the descriptions of the meaning attached to those experiences provided a more positive perspective. While recognising the struggle for being women living with HIV, they were more solution-focused on what it means to live with HIV in the social context. This means that if these studies are confirmed in other rural settings of Zimbabwe, they could help to inform healthcare provision, stigma and discrimination reduction interventions, and public health policy, and could, eventually, help to bring the global HIV epidemic under control as they will also help reduce mental health and physical illness, because, as Logie et al.^25^ posit, those who have experienced HIV-related stigma have had higher levels of psychological and physical condition.

## Conclusion

The meaning attached to the lived experiences of women living with HIV in rural Zimbabwe highlighted how women living with HIV viewed themselves regarding their accumulated lived experiences of being HIV-positive.

Women living with HIV in rural Zimbabwe viewed life as a perpetual struggle to maintain one’s humanness and quality of life. The battle to maintain humanness aims to overcome the loss of social belonging and reduced access to community-based empowerment opportunities. At the same time, the struggle to preserve the quality of life targeted the lack of need-based community healthcare and food insecurity attributed to their HIV-positive status. The researchers used these findings to develop a framework of the meaning attached to the lived experiences of women living with HIV in rural Zimbabwe (see Figure 1). This study’s results will support the efforts of the Zimbabwean government to improve the quality of life of HIV-positive women living in rural areas.

## References

[1] UNAIDS. Confronting inequalities – Lessons for pandemic responses from 40 years of AIDS. Global AIDS Update [homepage on the Internet]. Geneva; 2021 [cited n.d.]. Available from: https://www.unaids.org/en/resources/documents/2021/2021-global--aids-update

[2] Quinn TC. Forty years of AIDS: A retrospective and the way forward. J Clin Invest. 2021;131(18):e154196. 10.1172/JCI154196 34523618 PMC8439598

[3] Stangl AL, Earnshaw VA, Logie CH, et al. The health stigma and discrimination framework: A global, crosscutting framework to inform research, intervention development, and policy on health-related stigmas. BMC Med. 2019;17(1):31. 10.1186/s12916-019-1271-3 30764826 PMC6376797

[4] NAC Zimbabwe. 2018 HIV estimates fact sheet HIV prevalence [homepage on the Internet]. NAC Zimbabwe; 2018 [cited n.d.]. Available from: https://www.nac.org.zw/wp-content/uploads/2021/04/Zimbabwe-HIV-and-AIDS-2018-Estimates-Fact-Sheet.pdf

[5] Logie CH, Wang Y, Lacombe-Duncan A, et al. HIV-related stigma, racial discrimination, and gender discrimination: Pathways to physical and mental health-related quality of life among a national cohort of women living with HIV. Prev Med. 2018;107:36–44. 10.1016/j.ypmed.2017.12.018 29277410

[6] Brinsdon A, Abel G, Desrosiers J. ‘I’m taking control’: How people living with HIV/AIDS manage stigma in health interactions. AIDS Care. 2017;29(2):185–188. 10.1080/09540121.2016.1204420 27376836

[7] Panda S, Das RS, Maruf SKA, Pahari S. Exploring stigma in low HIV prevalence settings in rural West Bengal, India: Identification of intervention considerations. J Mix Methods Res. 2015;9(4):362–385. 10.1177/1558689814535843

[8] Mhode M, Nyamhanga T. Experiences and impact of stigma and discrimination among people on antiretroviral therapy in Dar es Salaam: A qualitative perspective. AIDS Res Treat. 2016;2016:7925052. 10.1155/2016/7925052 27110395 PMC4823479

[9] Ncube G. Eternal mothers, whores or witches: The oddities of being a woman in politics in Zimbabwe. Agenda. 2020;34(4):25–33. 10.1080/10130950.2020.1749523

[10] Halli SS, Khan CGH, Moses S, et al. Family and community level stigma and discrimination among women living with HIV/AIDS in a high HIV prevalence district of India. J HIV AIDS Soc Serv. 2017;16(1):4–19. 10.1080/15381501.2015.1107798

[11] Barlett CP. Predicting cyberbullying: Research, theory, and intervention [homepage on the Internet]. Washington, DC: Elsevier Academic Press; 2019 [cited n.d.]. Available from: https://psycnet.apa.org/record/2019-32176-000

[12] Ham M, Jeger M, Ivković AF. The role of subjective norms in forming the intention to purchase green food. Econ Res Ekon Istraz. 2015;28(1):738–748. 10.1080/1331677X.2015.1083875

[13] Creswell J. Steps in conducting a scholarly mixed methods study [homepage on the Internet]. Digital commons @ University of Nebraska-Lincoln; 2014 [cited n.d.]. Available from: https://digitalcommons.unl.edu/cgi/viewcontent.cgi?article=1047&context=dberspeakers

[14] Creswell J. A concise introduction to mixed methods research [homepage on the Internet]. California: Sage Publications.Inc; 2015 [cited n.d.];1999. Available from: https://www.manaraa.com/upload/d11df289-14cd-482b-a413-54c290668e4b.pdf

[15] Cuthbertson LM, Robb YA, Blair S. Theory and application of research principles and philosophical underpinning for a study utilising interpretative phenomenological analysis. Radiography. 2020;26(2):e94–e102. 10.1016/j.radi.2019.11.092 32052765

[16] Christensen LB, Johnson RB, Turner LA. Research methods, design, and analysis (Global ed.). Alabama: University of South Alabama; 2015.

[17] Morrissey G, Higgs J. Phenomenological research and adolescent female sexuality: Discoveries and applications. Qual Rep. 2006;11(1):161–168.

[18] Polit DF, Dan Beck CT. Resource manual for nursing research: Generating and assessing of evidence for nursing practice. 9th ed. Philadelphia: Lippincott, Williams & Wilkins; 2012.

[19] Van Manen M. Phenomenology of practice: Meaning-giving methods in phenomenological research and writing (Developing Qualitative Inquiry). Walnut Creek, CA: Left Coast Press; 2014.

[20] Matsena-Zingoni Z, Chirwa T, Todd J, Musenge E. Loss to follow-up risk among HIV patients on ART in Zimbabwe, 2009–2016: Hierarchical Bayesian spatio-temporal modeling. Int J Environ Res Public Health. 2022;19(17):11013. 10.3390/ijerph191711013 36078729 PMC9518110

[21] Lentoor AG. Caregiving and experiences of health, illness and coping in the context of paediatric and adolescent HIV and poverty. In: Mollaoglu M, editor. Caregiving and home care. IntechOpen, 2018; p. 147. 10.5772/intechopen.68759

[22] Ndlovu T, Mjimba V. Drought risk-reduction and gender dynamics in communal cattle farming in southern Zimbabwe. Int J Disaster Risk Reduct. 2021;58:102203. 10.1016/j.ijdrr.2021.102203

[23] Mwambene L. Recent legal responses to child marriage in Southern Africa: The case of Zimbabwe, South Africa and Malawi. Afr Hum Rights Law J. 2018;18(2):527–550. 10.17159/1996-2096/2018/v18n2a5

[24] Mahamboro DB, Fauk NK, Ward PR, Merry MS, Siri TA, Mwanri L. HIV stigma and moral judgment: Qualitative exploration of the experiences of HIV stigma and discrimination among married men living with HIV in Yogyakarta. Int J Environ Res Public Health. 2020;17(2):636. 10.3390/ijerph17020636 31963807 PMC7013688

[25] Logie CH, James Ll, Tharao W, Loutfy MR. HIV, gender, race, sexual orientation, and sex work: A qualitative study of intersectional stigma experienced by HIV-positive women in Ontario, Canada. PLoS Med. 2011;8(11):e1001124. 10.1371/journal.pmed.1001124 22131907 PMC3222645

[26] UN Women. Ending violence against women in the Western Balkans and Turkey: Implementing norms, changing minds [homepage on the Internet]. Final evaluation report. United Nations UN Women HQ; 2020 [cited n.d.]. Available from: https://eca.unwomen.org/en/digital-library/publications/2020/05/evaluation-brief-of-the-un-womens-regional-programme-ending-violence-against-women

[27] Goodwin T, Gregson S, Maswera R, Moorhouse L, Nyamukapa C. Understanding the determinants and consequences of HIV status disclosure in Manicaland, Zimbabwe: Cross-sectional and prospective analyses. AIDS Care. 2021;33(12):1577–1594. 10.1080/09540121.2021.1883507 33813969

[28] Fauk NK, Hawke K, Mwanri L, Ward PR. Stigma and discrimination towards people living with HIV in the context of families, communities, and healthcare settings: A qualitative study in Indonesia. Int J Environ Res Public Health. 2021;18(10):5424. 10.3390/ijerph18105424 34069471 PMC8159085

[29] Verhey R, Chibanda D, Vera A, Manda E, Brakarsh J, Seedat S. Perceptions of HIV-related trauma in people living with HIV in Zimbabwe’s Friendship Bench Program: A qualitative analysis of counselors’ and clients’ experiences. Transcult Psychiatry. 2020;57(1):161–172. 10.1177/1363461519850337 31180824

